# Novel Use of Glucagon-Like Peptide-1 (GLP-1) and Dual Glucose-Dependent Insulinotropic Polypeptide (GIP)/GLP-1 Receptor Agonists in Maturity-Onset Diabetes of the Young (MODY)

**DOI:** 10.7759/cureus.94882

**Published:** 2025-10-18

**Authors:** Abdalla Hilal, Bachar Afandi, Raya Almazrouei

**Affiliations:** 1 Division of Academic Affairs, Department of Internal Medicine, Tawam Hospital, Al Ain, ARE; 2 Division of Endocrinology, Department of Internal Medicine, Tawam Hospital, Al Ain, ARE; 3 Department of Internal Medicine, College of Medicine and Health Sciences, United Arab Emirates University, Al Ain, ARE

**Keywords:** dual gip/glp-1 agonist, glp-1 receptor agonist, hnf1a, maturity-onset diabetes of the young (mody), monogenic diabetes, tirzepatide

## Abstract

Introduction

Maturity-onset diabetes of the young (MODY), particularly due to HNF1A variants, is usually treated with sulfonylureas or insulin. In patients with excess weight or poor glycaemic control, these approaches can be challenging. Incretin-based therapies may provide a safer and more effective alternative.

Methods

We conducted a retrospective case series at Tawam Hospital (2019-2024), including patients with genetically confirmed MODY who received a Glucagon-like peptide-1 (GLP-1) receptor agonist or dual GLP-1/Glucose-dependent insulinotropic polypeptide (GIP) receptor agonist for at least three months. Clinical data, including HbA1c, body mass index (BMI), and insulin use, were extracted from medical records. The primary outcome was change in HbA1c; secondary outcomes included changes in BMI and insulin requirements.

Results

Six patients were included (five with HNF1A variants, one with combined PAX4/PDX1 variants). At baseline, HbA1c ranged from 7.3% to 10.6% and BMI from 25.1 to 36.7 kg/m². Following treatment, HbA1c improved in all cases (reductions of 1.0-4.1 percentage points) and weight decreased by 2.6-29 kg. Three of four insulin-treated patients discontinued insulin, while insulin-naïve patients maintained good glycaemic control without insulin initiation.

Conclusion

GLP-1 receptor agonists and dual GIP/GLP-1 receptor agonists improved glycaemic control, reduced body weight, and decreased insulin dependency in MODY patients. These findings suggest incretin-based therapies may be a safe and effective option in selected genotypes, meriting further evaluation in prospective studies.

## Introduction

Maturity-onset diabetes of the young (MODY) is a clinically heterogeneous group of autosomal-dominant, monogenic disorders that present with early-onset, non-autoimmune hyperglycaemia. Accurate molecular diagnosis is clinically actionable because genotype-phenotype patterns often influence prognosis, family screening, and treatment choices [1].

Monogenic diabetes requires treatment strategies informed by its underlying pathophysiological basis. In subtypes such as HNF1A-, HNF4A-, HNF1B-, and ABCC8-MODY, the primary defect involves impaired coupling of glucose sensing to insulin secretion in pancreatic β-cells, a mechanism that differs fundamentally from the insulin resistance typical of type 2 diabetes. Incretin-based therapies align well with this physiology: Glucagon-like peptide-1 receptor agonists (GLP-1 RAs) enhance intracellular cyclic adenosine monophosphate (cAMP) signaling in β-cells, amplify glucose-dependent insulin secretion, and suppress inappropriate glucagon release. When combined with Glucose-dependent insulinotropic polypeptide (GIP) receptor agonists (GLP-1/GIP RAs), these agents exert complementary islet effects and often promote greater weight loss, all with a substantially lower risk of hypoglycaemia than sulfonylureas [2]. This mechanistic alignment makes GLP-1-based therapies particularly attractive in the management of select MODY subtypes.

What was once theoretical is now increasingly supported by real-world evidence. In HNF4A-MODY, for instance, a father and son transitioned from suboptimal control to HbA1c values in the mid-6% and high-5% range with GLP-1 RAs), accompanied by fewer hypoglycaemic events [3]. In HNF1A- and HNF4A-MODY, dual GLP-1/GIP RAs such as tirzepatide have demonstrated robust reductions in both HbA1c and weight, while enabling reduction or discontinuation of sulfonylureas and insulin [4]. Similarly, in ABCC8-MODY, weekly semaglutide has facilitated complete insulin withdrawal with improved glycaemia and weight loss, a result that is physiologically plausible given GLP-1’s ability to stimulate exocytosis downstream of impaired ATP-sensitive potassium channel function [5]. The largest report to date, from two specialist centres, found that in HNF1A/HNF4A patients, GLP-1 RAs reduced mean HbA1c by 1.3%, BMI by 2.9 kg/m², and sulfonylurea dose by about two-thirds. With dual agonists, the weight effects were even more striking and some patients stopped insulin altogether [6].

Despite these encouraging reports, published data remain limited, particularly for rare MODY genotypes such as homozygous HNF1A or combined variants involving PAX4 and PDX1. Here we share our own experience using GLP-1 RA and dual GIP/GLP-1 RA therapy in genetically confirmed MODY, with a focus on glycaemic control, weight, and the potential to reduce or stop sulfonylureas and insulin.

## Materials and methods

This retrospective study was conducted at Tawam Hospital, a tertiary referral centre located in Al Ain, United Arab Emirates. We reviewed all patients with genetically confirmed MODY who received treatment with a GLP-1 RA or a dual GLP-1/GIP RA between January 2019 and December 2024. Patients were eligible for inclusion if they carried a pathogenic or likely pathogenic variant in a monogenic diabetes-related gene and had been treated with a GLP-1-based therapy for at least three months. Individuals with incomplete clinical records or insufficient follow-up data were excluded.

In total, seven cases were identified; one was excluded because of insufficient follow-up. Clinical data were extracted from electronic medical records, including age, sex, age at diabetes diagnosis, family history, genetic findings, presence of complications, baseline and follow-up weight, BMI, and glycated haemoglobin (HbA1c). Treatment details were recorded before and after GLP-1 therapy, including background insulin and oral hypoglycaemic use.

The primary outcome was the change in HbA1c. Secondary outcomes included changes in body weight and BMI, and the ability to discontinue or reduce insulin therapy. Given the small sample size, the analysis was descriptive. The study was approved by the Al Ain Research Ethics Committee (approval no. MF2058-2025-1291). As this was a retrospective review of anonymised data, the requirement for informed consent was waived.

## Results

Six patients were included in the study, five with HNF1A-MODY (four heterozygous, one homozygous) and one with double heterozygous PAX4/PDX1 variants (variant of uncertain significance, both predicted to be deleterious). The mean age at inclusion was 40 years (range 26-52), and the mean age at onset of diabetes was 16 years (range eight to 25). At baseline, HbA1c ranged from 7.3% to 10.6% and BMI from 25.1 to 36.7 kg/m². Four patients were receiving insulin therapy, while two were insulin-naïve.

Overall outcomes are summarised in Table 1.

**Table 1:** Clinical characteristics and outcomes of MODY cases treated with GLP-1-based therapies M: Male patient; F: Female patient; MODY: Maturity onset diabetes of the young; GLP-1: Glucagon-like peptide-1; GIP: Glucose-dependent insulinotropic polypeptide; KT: Kidney transplant; PDR: Proliferative Diabetic Retinopathy; MI: Myocardial infarction; PVD: Peripheral vascular disease; Dapa: Dapagliflozin; VUS: Variant of uncertain significance; ↓: reduction; het: heterozygous; homo: homozygous. Arrows (→) indicate dose escalation; Outcomes reported after ≥3 months on therapy.

Case	Age/Sex	Onset age	Genetic variant	Complications	Pre-therapy	Baseline HbA1c	Baseline BMI	GLP-1/GIP agent	GLP-1/GIP agent duration before reported outcome	Outcome
1	52F	14y	HNF1A (het, (c.864delinsCC, p.Gly292Argfs*25)	Micro+macro (MI 47y)	Gliclazide 120 mg, Metformin 1 g daily, Dapa 10 mg daily, Insulin degludec 18 units daily.	7.50%	32.4	Tirzepatide 2.5→5 mg	4 months	HbA1c 6.3%, ↓9 kg, insulin stopped
2	44M	18y	HNF1A (het, (c.864delinsCC, p.Gly292Argfs*25)	Nephropathy (KT), PDR+glaucoma, PVD	Basal-bolus insulin (glargine 20 units and lispro 6 units at meal times), Metformin 500 mg twice daily.	9.60%	36.5	Tirzepatide 2.5→5 mg	16 months	HbA1c 5.5–5.8%, ↓29 kg, insulin stopped
3	34F	8y	HNF1A (het, (c.864delinsCC, p.Gly292Argfs*25)	None	Basal-bolus insulin (glargine 16 units, aspart 12 units at meal times)	7.30%	36.7	Tirzepatide 2.5→7.5 mg	8 months	HbA1c 5.7%, ↓20 kg, insulin stopped
4	26F	18y	PAX4 (het, (c.199A>C, p.Ser67Arg) + PDX1 (het, (c.409G>T, p.Gly137Cys)(VUSs)	None	Dapa 10mg daily , Glyburide/Metformin 2.5/500 mg twice daily	10.60%	25.1	Semaglutide 0.25→0.5 mg	3 months	HbA1c 7.1%, ↓2.6 kg
5	41F	15y	HNF1A (homo, c.1136C>G, p.Pro379Arg)	None	Basal-bolus insulin (insulin detemir 24 units daily, insulin aspart 14 units meal times) + Gliclazide 60mg daily	8.40%	28	Semaglutide 0.25→1 mg	8 months	HbA1c 6.7%, ↓7.1 kg, insulin continued
6	43F	25y	HNF1A (het, (c.864delinsCC, p.Gly292Argfs*25)	None	Metformin 500 mg three times daily, gliclazide 60 mg daily, empagliflozin 25 mg daily	9.90%	36.7	Dulaglutide 1.5 mg	4 months	HbA1c 6.8%, ↓9.4 kg; stable with semaglutide/tirzepatide

Following GLP-1-based therapy, HbA1c improved in all patients, with absolute reductions of 1.0-4.1 percentage points. Body weight decreased consistently across the series, with losses ranging from 2.6 to 29 kg. Among the four patients on insulin at baseline, three discontinued insulin completely, whereas one (with homozygous HNF1A) remained insulin-dependent. Both insulin-naïve patients maintained glycaemic improvement without requiring insulin initiation.

## Discussion

Our series stands out for three reasons. First, it includes four patients who all carried the same pathogenic HNF1A frameshift (c.864delinsCC; p.Gly292Argfs*25), but were at very different stages of disease and complication burden. Until now, only a single patient treated with a GLP-1 RA and carrying this variant has been described [7]. Second, it features a patient with homozygous HNF1A p.Pro379Arg, an exceptionally rare genotype. To our knowledge, this is the first report of incretin therapy in such a case [8]. Third, it describes a patient carrying two predicted deleterious variants in different MODY genes (PAX4 p.Ser67Arg and PDX1 p.Gly137Cys). No previous GLP-1 RA or dual agonist cases have been published in this context, and the role of PAX4 as a MODY gene remains debated [9,10].

Mechanistic understanding and clinical experience point in the same direction. Because incretin action is glucose-dependent, these drugs improve blood glucose control without provoking hypoglycaemia - a persistent problem for many patients treated with sulfonylureas and, later, insulin. At the cellular level, activation of the cAMP-protein kinase A/exchange protein activated by cAMP (PKA/Epac) pathway provides an alternative route to stimulate insulin secretion, effectively bypassing the adenosine triphosphate (ATP)-sensitive potassium channel defects. This helps explain why good responses have also been observed in ABCC8-MODY [2].

One of the clearest early examples came from HNF4A-MODY: in a single family, semaglutide and liraglutide both produced substantial HbA1c improvements, fewer hypoglycaemic events, and a simpler treatment routine [3]. In both HNF1A- and HNF4A-MODY, tirzepatide has since been used in routine practice to achieve better glucose and weight outcomes, while allowing patients to reduce or discontinue other agents [4]. This pattern was reinforced in a two-centre case series, where GLP-1 RA lowered HbA1c and BMI and substantially reduced sulfonylurea requirements, while dual agonists provided an additional weight benefit and, in some cases, enabled insulin withdrawal [6].

In HNF1A-MODY, weekly GLP-1 RA monotherapy has maintained excellent glycaemic control without hypoglycaemia, offering a clear alternative when sulfonylureas lead to weight gain or recurrent hypoglycaemic episodes [11]. For patients reluctant to use injections, oral semaglutide has provided another option, reducing sulfonylurea requirements, lowering HbA1c into the 5% range, and achieving double-digit weight loss within six months [12]. Even in HNF1B-MODY, where insulin is often the default, incretin therapy has shown promise: semaglutide has enabled insulin withdrawal, improved time-in-range, and modest weight loss, while tirzepatide has increased fasting C-peptide and dramatically reduced insulin needs [13,14]. In ABCC8-MODY, by acting downstream of the defective potassium channel, semaglutide has allowed complete insulin cessation with sustained improvements in HbA1c, weight, and stimulated C-peptide (5).

From a practical standpoint, three themes stand out. First, the glucose-dependent action of incretins means a much lower risk of hypoglycaemia - a major advantage for patients with active lifestyles or fluctuating intake [11]. Second, weight loss is consistent with GLP-1 RA and often greater with dual agonists, which is particularly valuable when excess weight compounds the metabolic burden [4]. Third, these drugs can simplify treatment - reducing or eliminating sulfonylureas and insulin, and in some cases allowing patients to transition to a once-weekly regimen [6].

Tolerability remains the main consideration. Nausea is common during the early weeks of therapy but usually improves with gradual dose titration and simple advice on eating patterns and hydration. For patients hesitant about injections, oral semaglutide offers an effective alternative, provided they can adhere to the specific dosing routine [12]. When weight loss and insulin-sparing are major priorities, or when GLP-1 RA alone proves insufficient, dual GIP/GLP-1 RA therapy represents a logical next step, with careful dose escalation.

This study has several limitations. The number of cases was small and the design retrospective, limiting generalizability and precluding statistical inference. Follow-up durations varied, and long-term durability of glycaemic and weight outcomes remains uncertain. Genetic diversity was limited to a few subtypes, with over-representation of HNF1A-MODY, meaning the results cannot be extrapolated to all forms of monogenic diabetes. Finally, patient-reported outcomes such as quality of life, treatment satisfaction, and hypoglycaemia burden were not systematically captured.

## Conclusions

In conclusion, our experience adds to a growing body of evidence that GLP-1 RAs and dual GIP/GLP-1 RAs can be safe and effective in selected MODY patients. Across six cases, these therapies consistently lowered HbA1c, promoted clinically meaningful weight loss and, in most patients, reduced or eliminated the need for insulin. The series also highlights novel genetic contexts - including homozygous HNF1A and combined PAX4/PDX1 variants - in which incretin therapy has not been described previously. Taken together, these findings suggest that GLP-1-based therapies offer a rational and patient-friendly option in monogenic diabetes, meriting further evaluation in larger, prospective studies stratified by genotype.
